# Supplementary figures and images for: The Role of Nuclear Factor of Activated T Cells 5 in Hyperosmotic Stress-Exposed Human Lens Epithelial Cells
Source: Int J Mol Sci. 2021 Jun 11;22(12):6296. doi: 10.3390/ijms22126296 (PMC8230750; doi:10.3390/ijms22126296)

**A**

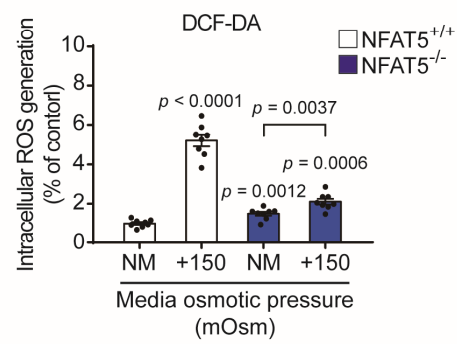

**A**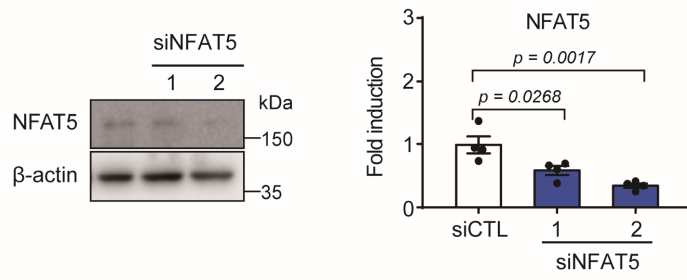**B**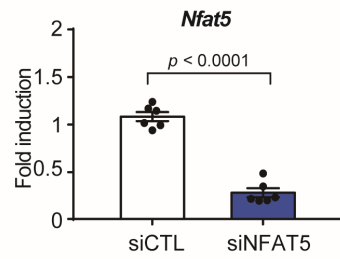**C**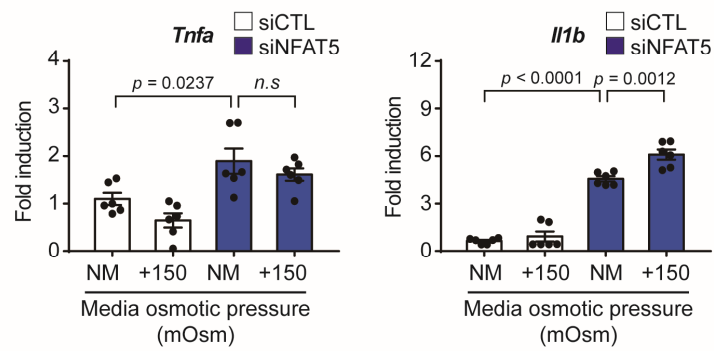**D**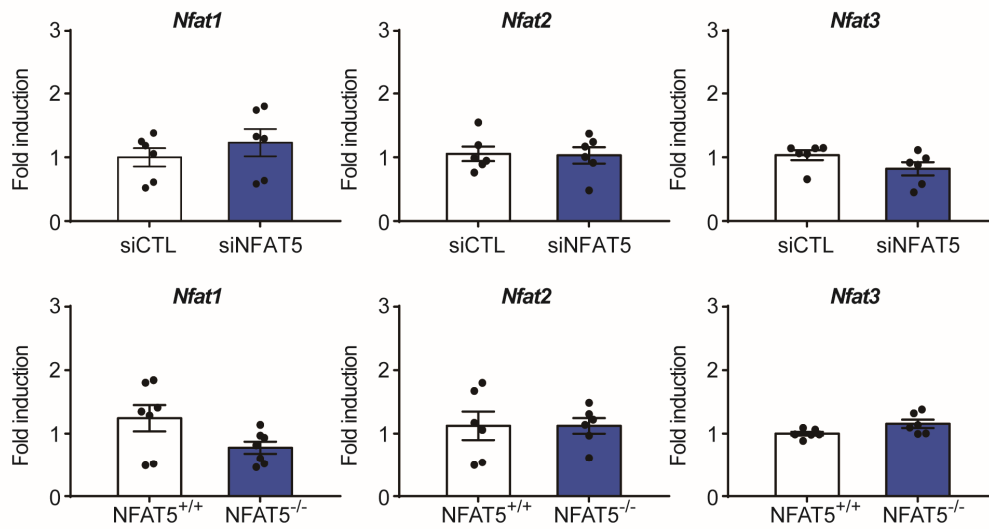

Supplementary Figure 2

Supplement: Supplementary file 1 [file ijms-22-06296-s001.zip › ijms-1235476-supplementary.pdf]
